# MiRNA‐224‐5p regulates the defective permeability barrier in sensitive skin by targeting claudin‐5

**DOI:** 10.1111/srt.13720

**Published:** 2024-05-14

**Authors:** Li Yang, Wen‐Juan Wu, Le‐Chun Lyu, Ying Tu, Hua Gu, Xiang‐Feng Chen, Yan‐Jie Chai, Mao‐Qiang Man, Li He

**Affiliations:** ^1^ Department of Dermatology First Affiliated Hospital of Kunming Medical University, Institute of Dermatology & Venereology of Yunnan Province Kunming China; ^2^ Department of Dermatology People's Hospital of Henan Province Zhengzhou China; ^3^ Department of Physiology Kunming Medical University Kunming China; ^4^ Dermatology Service Veterans Affairs Medical Center and Department of Dermatology University of California San Francisco USA; ^5^ Skin Health Research Center Yunnan Characteristic Plant Extraction Laboratory Kunming China

**Keywords:** claudin‐5, miR‐224‐5p, permeability barrier, sensitive skin, tight junction

## Abstract

**Background:**

Sensitive skin is hypersensitive to various external stimuli and a defective epidermal permeability barrier is an important clinical feature of sensitive skin. Claudin‐5 (*CLDN5*) expression levels decrease in sensitive skin. This study aimed to explore the impact of *CLDN5* deficiency on the permeability barrier in sensitive skin and the regulatory role of miRNAs in *CLDN5* expression.

**Materials and methods:**

A total of 26 patients were retrospectively enrolled, and the CLDN5 expression and permeability barrier dysfunction in vitro were assessed. Then miRNA‐224‐5p expression was also assessed in sensitive skin.

**Results:**

Immunofluorescence and electron microscopy revealed reduced *CLDN5* expression, increased miR‐224‐5p expression, and disrupted intercellular junctions in sensitive skin. *CLDN5* knockdown was associated with lower transepithelial electrical resistance (TEER) and Lucifer yellow penetration in keratinocytes and organotypic skin models. The RNA‐seq and qRT‐PCR results indicated elevated miR‐224‐5p expression in sensitive skin; MiR‐224‐5p directly interacted with the 3`UTR of *CLDN5*, resulting in *CLDN5* deficiency in the luciferase reporter assay. Finally, miR‐224‐5p reduced TEER in keratinocyte cultures.

**Conclusion:**

These results suggest that the miR‐224‐5p‐induced reduction in *CLDN5* expression leads to impaired permeability barrier function, and that miR‐224‐5p could be a potential therapeutic target for sensitive skin.

## 1 INTRODUCTION

Sensitive skin mainly manifests on the facial area. When exposed to physical, chemical, and psychological stimuli, individuals often experience subjective sensations such as burning, stinging, itching, and tightness.^1^, ^2^, ^3^ Sensitive skin affects nearly 50% of all people, including 60% of women and 40% of men. The prevalence of sensitive skin varies across countries and has increased over the past few decades.^1^, ^4^ Sensitive skin is usually determined via sensory testing methods, such as stinging tests with lactic acid, capsaicin, or dimethyl sulfoxide.^5^ Although the pathogenesis of sensitive skin is unclear, a defective epidermal permeability barrier has been linked to the development of sensitive skin symptoms, because it can increase the transcutaneous penetration of water‐soluble chemicals and allergens, in addition to inducing cutaneous inflammation.^6^, ^7^

Tight junctions (TJs), junctional structures between epidermal cells, play an important role in maintaining a normal skin permeability barrier. TJs are composed of complex transmembrane proteins, including claudins, occludin, tricellulin, JAM, ZO‐1, 2, and 3, and cingulin, all of which are determinants of the epidermal permeability barrier. Our previous study revealed that the mRNA expression levels of epidermal claudin‐5 (CLDN5) were significantly decreased in sensitive skin.^8^ Studies have demonstrated that miR‐224‐5p is highly expressed in malignancies, especially colorectal cancer, diffuse large B‐cell lymphoma, and gastric cancer.^9^ Through bioinformatics analysis, we noted that miR‐224‐5p expression increased in sensitive skin, and that the target gene of miR‐224‐5p was *CLDN5*. However, whether miR‐224‐5p expression is linked to permeability barrier defects in sensitive skin by targeting CLDN5 remains unclear.

In the present study, we determined the link between CLDN5 deficiency and the permeability barrier in sensitive skin, as well as the regulatory role of miR‐224‐5p in CLDN5 expression.

## 2 MATERIALS AND METHODS

### 2.1 Subjects and samples

A total of 26 female patients (38.3 years) were enrolled from the First Affiliated Hospital of Kunming Medical University. Sensitive skin was diagnosed using a questionnaire (Supplementary Table S1) and a lactic acid stinging test.^10^, ^11^ The subjects who responded positive to a minimum of five out of seven questions and had stinging test scores ≥ 3 were defined as having sensitive skin. Patients with other dermatoses, including acne, pityriasis rosacea, contact dermatitis, eczema, and/or other skin diseases, were excluded. Skin tissue was obtained by trimming the area surrounding the nevus. The study protocol was approved by the Ethics Committee of First Affiliated Hospital of Kunming Medical University. Written informed consent was obtained from all participants.

### 2.2 RNA‐seq analysis

Total RNA was extracted from the facial skin samples of six subjects using the mirVanaTM miRNA isolation kit (Ambion, Austin) and further purified using an RNeasy column (QIAGEN, Hilden, Germany). Small RNA libraries were constructed and sequenced using Illumina. Total transcriptome libraries were prepared by the Novogene Bioinformatics Technology Cooperation (Beijing, China).

In silico analysis of small sequencing data and raw reads was performed using Illumina's Genome Analyzer Pipeline software. After trimming the sequencing adapters, the resulting reads were successively filtered by read length (only those ranging from 18 to 35 nt were retained). Subsequently, the retained reads were searched against NCBI to remove known human classes of RNAs. Sequencing reads that passed the strict filter rules were mapped to the human genome.

The expression levels and correlations between the miRNAs in each sample were determined and normalized using TPM.^7^ The DESeq2 program was employed to detect differentially expressed miRNAs in sensitive and normal skin.^12^

### 2.3 Quantitative real‐time PCR (qRT‐PCR)

Total RNA isolated from 10 sensitive skin and 10 normal facial tissue samples was reverse‐transcribed to cDNA using an All‐in‐One™ First‐Strand cDNA Synthesis Kit (Gene Copoeia) following the manufacturer's protocol. MiR‐224‐5p and its internal control U6, as well as the mRNA transcript (ENST00000413119) of CLDN5 and its internal control β‐actin were selected to validate the result. The 2^−ΔΔCt^ method was used with its internal control to relatively quantify the detected transcripts. The primer sequences are listed in Supplementary Table S2.

### 2.4 Plasmids

pCMV6‐CLDN5 and pCMV6 were purchased from Origene Company (www.origene.com).

### 2.5 Immunofluorescence

Skin sections were incubated with CLDN5 antibodies (Abcam, USA) following permeabilization with 0.1% Triton X‐100, blocked with 0.5% bovine serum albumin, and incubated with a secondary antibody. Counterstaining was performed via Hoechst staining (Life Technologies). Images were captured under a fluorescence microscope. The exposure time for the fluorescent images was 300 ms.

### 2.6 Transmission electron microscopy

Skin tissues were fixed in Karnovsky's fixative according to standard procedures and embedded in EPON resin. Ultrathin sections (0.07 mm) were cut, stained with uranyl acetate and lead citrate, observed under a JEOUL 1010 transmission electron microscope (Jeoul, Peabody, MA) at 60 kV, and photographed with a camera incorporated in the microscope.

### 2.7 Cell culture and small interfering RNA (siRNA) transfection

Normal human keratinocytes (KCs) and fibroblasts from pediatric foreskin were cultured as described previously.^13^ Both the negative control (NC) and human *CLDN5*‐targeting siRNA were synthesized by GenePharma (Shanghai, China). The siRNA sequences are listed in Supplementary Table S3.

Third‐passage KCs at 50%−60% confluence were transfected with siRNA using Lipofectamine 2000 (Invitrogen) according to the manufacturer's instructions, with modifications. Then, 5 mL OPTI‐MEM medium (Gibco) was mixed with 50 µL Lipofectamine 2000 and 130 µL of a 20 µM siRNA solution or the NC RNA solution. After incubation at room temperature for 30 min, the solution was added to 20 mL KGM (Lonza) and transferred to the KCs. The KCs were incubated for 24 h before seeding onto a fibroblast collagen gel, as described below.

### 2.8 Preparation of *CLDN5*‐knockdown organotypic skin cultures

After the KCs were attached for 24 h, the co‐cultures were lifted to an air‐liquid interface and cultured for an additional 1–2 weeks. The culture medium was changed every 2 days. A 2.5 mL suspension of collagen type I (BD Bioscience) containing 2.5 × 10^5^ fibroblasts and balanced using 10× fetal bovine serum (Gibco) was poured into each cell culture insert (3 µm pore size; BD Bioscience) and allowed to gel for 2 h at 37°C. Then, 1.5 × 10^6^ KCs transfected with either siRNA or NC were seeded onto each collagen gel. After overnight incubation at 37°C, the medium was removed from both the inserts and external wells, and 10 mL serum‐free KC‐defined medium, consisting of KGM without bovine pituitary extract and supplemented with 1.3 mM calcium (Sigma, Vienna, Austria), 10 µg/mL transferrin (Sigma), 50 µg/mL ascorbic acid (Sigma), and 0.1% bovine serum albumin (Sigma), was added to each external well. The KCs were allowed to form a multilayered epidermis for 7 days, and the medium was changed every second day.^14^

### 2.9 TEER measurement

KCs were seeded onto cell culture inserts (Corning, Corning, NY) 24 h after siRNA transfection and grown to confluence. After 48 h of incubation, transepithelial electrical resistance (TEER) was measured using a Millicell‐ERS Voltohmmeter (Millipore). Readings from each well were repeated three times.

### 2.10 FITC

On day 7 after initiation of the organotypic skin culture, 20 mL of Lucifer yellow (Sigma, 1 mM) was applied onto the stratum corneum of the organotypic skin samples and incubated at 37°C for 2 h. Organotypic skin samples were fixed in 3.7% formaldehyde and embedded in paraffin. Lucifer yellow penetration was assessed using fluorescence microscopy.

### 2.11 Western blot

After 48 h of transfection, cells were collected, and 50 mL RIPA lysis buffer (Thermo) with 1% protease inhibitor was added to every 5 × 10^5^ cells on ice for 30 min. After centrifugation at 12,000 rpm for 10 min at 4°C, the supernatant was collected to determine protein concentration using a BCA protein assay. The supernatant was boiled for 5–10 min and then stored at‐20°C. The extracted protein was subjected to sodium dodecyl sulfate‐polyacrylamide gel electrophoresis for 1 h, then transferred to a polyvinylidene fluoride membrane for 20 min, and sealed in milk solution for 2 h. With the addition of rabbit anti‐human CLDN5 (Abcam) and mouse anti‐human, the membrane was maintained overnight at 4°C. Subsequently, HRP‐labeled goat anti‐rabbit IgG antibody and HRP‐labeled rabbit anti‐mouse IgG antibody were added, and samples were incubated at 37°C. Chemiluminescence visualization was performed in the dark according to the instructions of the electrochemiluminescence color kit (Thermo).

### 2.12 Luciferase reporter assay

Base pairs containing the complete 3′UTRs of *CLDN5* or *CLDN5* with point mutations in the miR‐224‐5p recognition site were constructed. The 3`UTRs of *CLDN5* cDNA containing wild‐type or mutated miR‐224‐5p binding sequences were synthesized. The sequence was cloned into the SacI/XhoI restriction sites of the pGL3 luciferase control reporter vector (Promega) to generate the CLDN5 3`UTR reporter. A total of 5 × 10^5^ KCs stably transfected with miR‐224‐5p mimic or miR‐NC were seeded into 6‐well plates. Cells were transfected with 2.5 µg of either pGL3‐WT‐CLDN5 or pGL3‐MUT‐CLDN5 3`UTR reporter plasmid. In addition, 100 µg of Renilla luciferase expression plasmid was also transfected into the above cells as a reference control. Firefly and Renilla luciferase activities were measured using a dual‐luciferase reporter assay (Promega, E1910, WI) 48 h after transfection, according to the manufacturer's instructions. The relative luciferase activity was calculated as the firefly/Renilla fluorescence ratio.

### 2.13 Statistical analysis

Continuous data were expressed as means ± SEM, and the differences were assessed using an independent *t*‐test for comparison between two groups and a one‐way analysis of variance (ANOVA) for comparison among three or more groups. Further pairwise comparisons were performed using a *Q* test when ANOVA showed significant differences among the groups. Pearson's correlation analysis was used for the correlation analysis. All reported *P*‐values were two‐sided, and the inspection level was 0.05. Statistical analyses were conducted using IBM SPSS Statistics for Windows (SPSS, Version 26.0).

## 3 RESULTS

### 3.1 Sensitive skin exhibits decreased CLDN5 protein expression levels and disrupted intercellular structure

**FIGURE 1:**
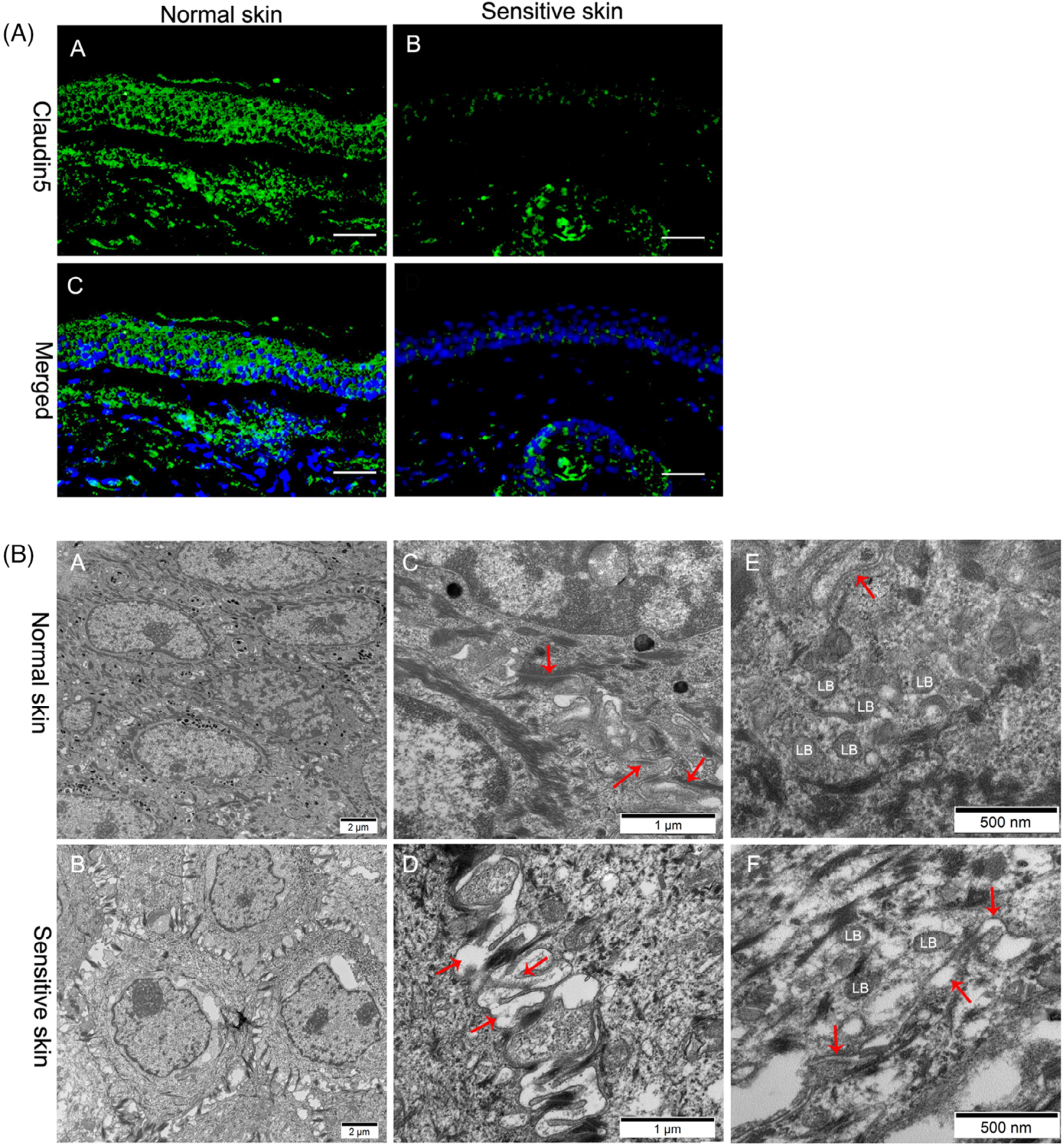
Immunofluorescence and electron microscopy of tissues. (A) Membranes and nuclei were stained with anti‐CLDN5 (green) and Hoechst (blue) in sensitive and normal skin, respectively. Scale bar = 50 nm. (B) Transmission electron microscopy. The TJ structure is shown in red arrows.

CLDN5 protein expression levels in normal and sensitive skin were assessed, and we noted that CLDN5 was almost completely diminished in sensitive skin (Figure 1A). In parallel, most of the intercellular space became empty in sensitive skin (Figure 1B (B, D, & F) vs. normal controls in Figure 1B (A,C, & E)). These results indicate that CLDN5 deficiency results in an altered intercellular structure in sensitive skin.

### 3.2 CLDN5 deficiency leads to permeability barrier dysfunction in vitro

**FIGURE 2:**
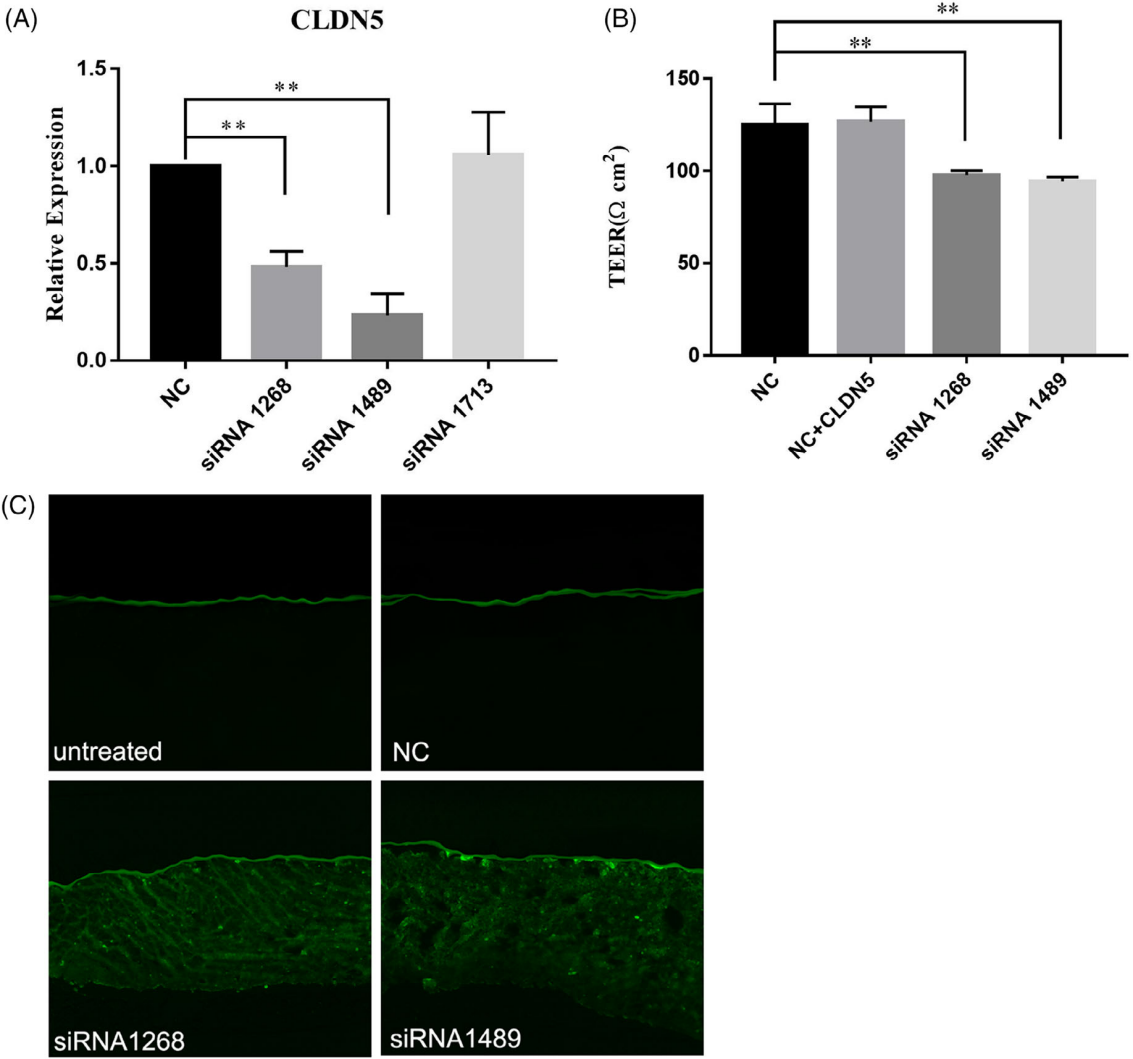
*CLDN5* downregulation impairs diffusion barrier function. (A) Relative CLDN5 expression levels after siRNA transfection. Data were normalized to NC (transfected with siRNA‐NC), set as 1 and shown in a dotted line. *P*‐values are versus normal controls (*n* = 3 for all). (B) *CLDN5* knockdown decreased TEER in KCs, compared to the NCs. Data were normalized to the NCs, set as 100% and shown in a dotted line. One‐way ANOVA was applied to assess the differences among groups. *P*‐values are versus normal controls (*n* = 3 for all). (C) Lucifer yellow fluorescence (green) was visualized in sections of organotypic skin cultures treated with either CLDN5 siRNA or siRNA‐NC.

TEER was applied to human KC cultures following treatment with siRNA to determine the functional consequence of CLDN5 deficiency, which is considered an indicator of permeability barrier function.^15^ As expected, both siRNAs 1268 and 1489 induced over 50% reduction in CLDN5 expression levels in comparison to the controls, accompanied by reduced TEER (Figure 2A,B). To further ascertain the role of CLDN5 in the permeability barrier, a Lucifer yellow penetration assay was performed using organotypic cultures of normal human KCs. As shown in Figure 2C, Lucifer yellow was mainly restricted to the surface of the organotypic skin. However, treatment with either siRNA 1268 or 1489 increased Lucifer yellow penetration into the epidermis (Figure 2C). Taken together, these results demonstrate that CLDN5 deficiency leads to a defective permeability barrier.

### 3.3 Elevation in miRNA‐224‐5p expression contributes to the reduced CLDN5 expression in sensitive skin

**FIGURE 3:**
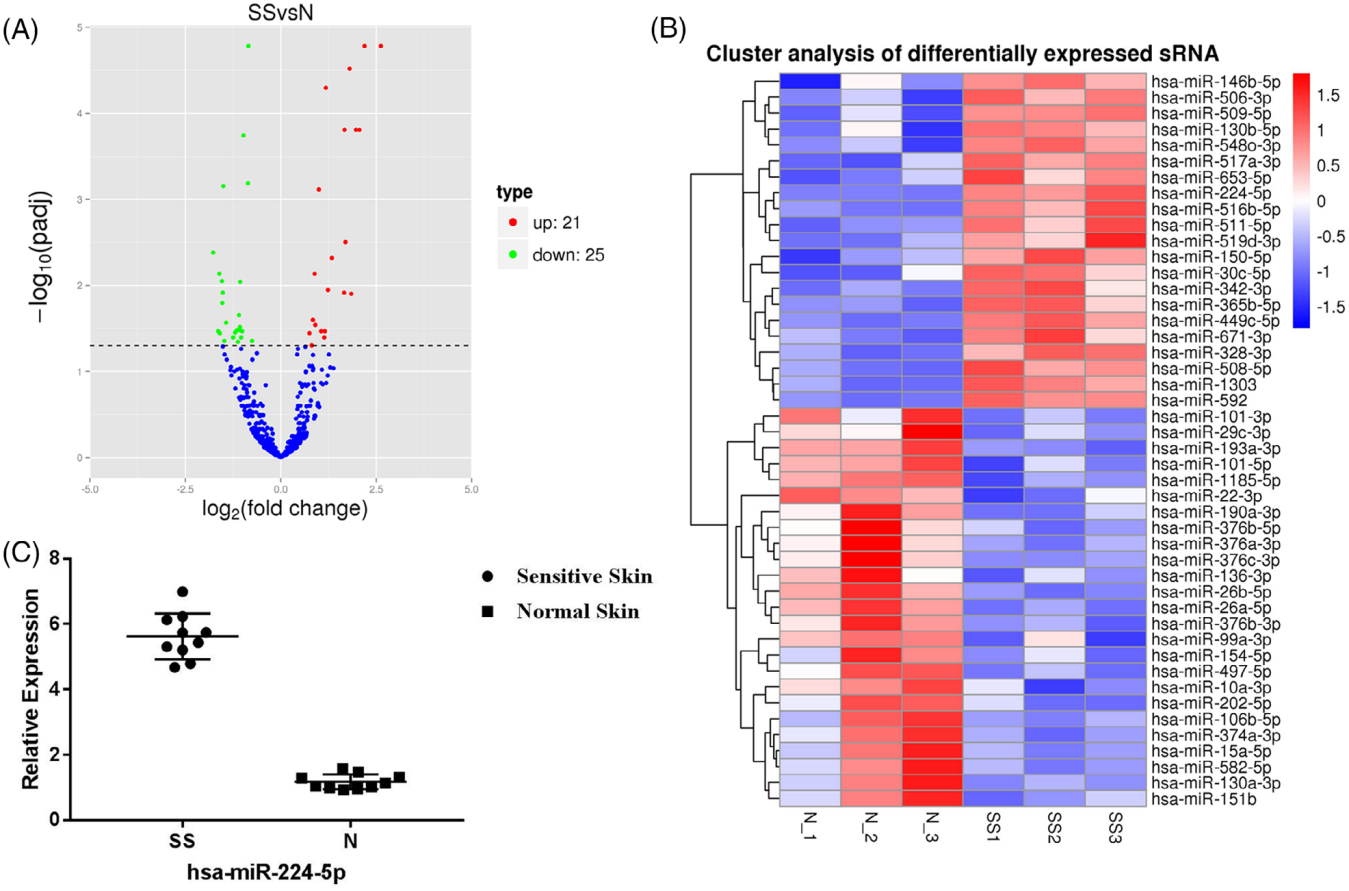
MiR‐224‐5p expression in sensitive and normal skin. (A) Volcano plots of DE transcripts. The differences in miRNA expression profiles can be noted in the overall distribution of the transcripts. The red points in the plot represent upregulated transcripts, and the green points represent downregulated transcripts. The default filter threshold is *P* < 0.05. (B) Hierarchical heat maps of the differentially expressed profiles in sensitive and normal skin. MiRNAs were used to analyze the gene expression data, where the cluster analysis arranged samples into groups according to their expression levels. Each column represents a sample, and each row represents a transcript. “Red” indicates higher expression, and “blue” indicates lower expression. SS, sensitive skin; N, normal skin. (C) Relative expression analysis of CLDN5 and miR‐224‐5p in sensitive and normal skin, as determined using qRT‐PCR (*n* = 10 for all).

First, we performed miRNA sequencing to determine the differential miRNA expression in sensitive and normal skin. Of the 46 differentially expressed miRNAs, 21 were upregulated, and 25 were downregulated in sensitive skin (Figure 3A). The top 20 differentially expressed miRNAs between sensitive and normal skin are shown in Supplementary Table S4. Heat maps and clustering of differentially expressed miRNAs were generated using unsupervised hierarchical clustering analysis (Figure 3B). Among the upregulated miRNAs, miRNA‐224‐5p was significantly upregulated in sensitive skin (*P* < 0.05 vs. normal skin, log_2_ fold‐change = 2.0595). To validate the RNA‐seq results, miRNA‐224‐5p expression levels were evaluated using qRT‐PCR. Indeed, sensitive skin exhibited an over 3‐fold increase in miRNA‐224‐5p expression compared to that in normal controls (Figure 3C). Further analysis using the miRanda software suggested that *CLDN5* is the target gene of miRNA‐224‐5p.

**FIGURE 4:**
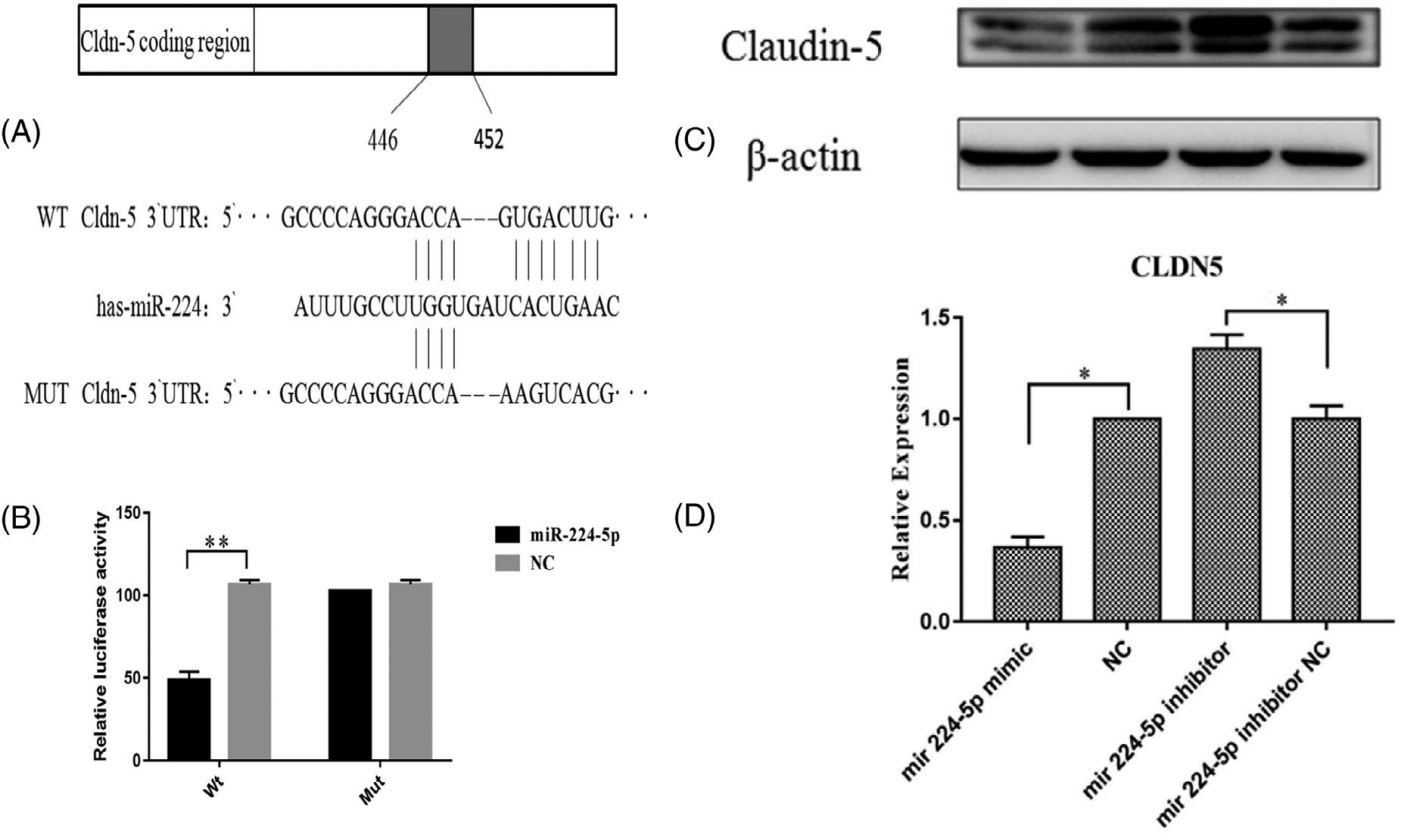
Effects of miR‐224‐5p on the 3`UTR of *CLDN5*. (A) MiR‐224 binding with the *CLDN5* 3`UTR region, and design of the 3`UTR *CLDN5* mutant site. (B) Relative luciferase activity of *CLDN5* wild type or mutant 3`UTR in KCs following transfection with the miR‐224‐5p mimic. One‐way ANOVA was used to determine significant differences. *P‐*values versus normal controls. (C) CLDN5 protein expression levels. Unpaired *t*‐test with Welch's correction was used to determine significant differences. *P*‐values are versus normal controls (*n* = 3 for all). (D) Western blotting shows changes in CLDN5 expression following treatment with the miR‐224‐5p mimic and miR‐224‐5p inhibitor (*n* = 3 for all).

A luciferase reporter assay was performed to further determine whether CLDN5 is the target gene of miR‐224‐5p. As expected, co‐transfection with miR‐224‐5p in KCs dramatically attenuated *CLDN5*‐3′UTR (Wt‐miR‐224‐5p/CLDN5)‐driven luciferase activity (*P* < 0.05), whereas the miR‐224‐5p mimic did not inhibit the luciferase activity driven by the *CLDN5*‐3′UTR with the mutation (Mut‐miR‐224‐5p/CLDN5) in the miR‐224‐5p recognition site (Figure 4A,B), suggesting that miR‐224‐5p directly targets the *CLDN5* gene.

To assess the regulatory role of miR‐224‐5p in CLDN5 expression, CLDN5 mRNA and protein expression levels were measured via qPCR and western blotting in KCs following treatment with miR‐224‐5p. MiR‐224‐5p dramatically reduced both mRNA (Figure 4C) and protein (Figure 4D) CLDN5 expression. Importantly, the miR‐224‐5p inhibitor overrode the inhibitory effect of miR‐224‐5p on CLDN5 expression (Figure 4C,D), indicating that miR‐224‐5p specifically targets CLDN5. Collectively, these results demonstrated that elevated miR‐224‐5p expression contributes to a reduction in CLDN5 expression.

### 3.4 MiR‐224‐5p‐induced reduction in CLDN5 expression leads to permeability barrier defects

**FIGURE 5:**
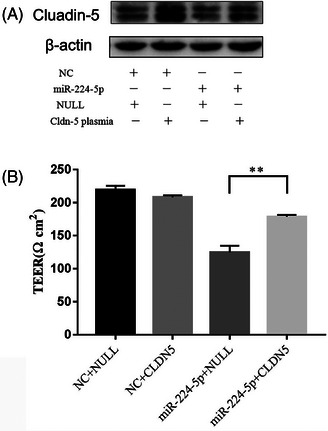
Comparisons of CLDN5 protein expression and TEER in the KCs among groups 48 h after transfection. (A) CLDN5 protein expression. (B) TEER. *P < 0.05. (*n* = 3 for all).

To elucidate the link between miR‐224‐5p and *CLDN5* in regulating the permeability barrier, TEER was measured in KC cultures following miR‐224‐5p mimic treatment with or without *CLDN5* overexpression. As shown in Figure 5A, the miR‐224‐5p mimic markedly reduced CLDN5 expression (first lane vs. third lane), whereas KC transfection with the CLDN5 plasmid increased CLDN5 expression (first lane vs. second lane). Moreover, miR‐224‐5p mimic‐treated KC co‐transfection with the CLDN5 plasmid normalized the CLDN5 expression levels (Figure 5A, third lane vs. last lane). Parallel to the reductions in CLDN5 expression, the miR‐224‐5p mimic reduced TEER in KC cultures. Transfection with the CLDN5 plasmid normalized TEER in cells treated with the miR‐224‐5p mimic (Figure 5B). These results demonstrate that miR‐224‐5p‐induced reduction in CLDN5 expression leads to a defective permeability barrier.

## 4 DISCUSSION

Although sensitive skin is not a life‐threatening condition, it can negatively affect patient quality of life.^16^ Managing sensitive skin is a challenge for both clinicians and patients, largely because of the uncertainty of its pathogenesis. Studies have demonstrated that the stratum corneum of sensitive skin is thinner with smaller and immature corneocytes.^17^, ^18^ A defective epidermal permeability barrier is significantly associated with skin sensitivity, and claudin proteins are required for its integrity.^7^, ^19^ CLDN5 mRNA expression levels were significantly lower in sensitive skin than those in normal skin.^8^ This study was performed to assess the role of CLDN5 deficiency in the permeability barrier in sensitive skin and the regulatory role of miRNAs in CLDN5 expression. We found that sensitive skin was associated with reduced CLDN5 expression, elevated miR‐224‐5p expression, and disrupted intercellular junctions, compared to normal skin. TEER and Lucifer yellow penetration in KCs and the organotypic skin model were reduced after *CLDN5* knockdown. A luciferase reporter assay showed that miR‐224‐5p directly interacted with the 3`UTR of *CLDN5*, negatively correlated with its expression, and reduced TEER in KC cultures. These results suggest that the miR‐224‐5p‐induced reduction in CLDN5 expression leads to impaired permeability barrier function, and that miR‐224‐5p could be a potential therapeutic target for sensitive skin.

TJs are intercellular contacts that seal the spaces between individual cells in an epithelial sheet. Compared with other junctions, TJs are composed of approximately 40 different proteins, including CLDN and ZO, both of which are important for epidermal permeability barrier function.^20^ Moreover, the regulatory roles of occludin in TJ formation and KC proliferation and differentiation have been demonstrated.^21^ Our previous studies revealed that the TJ protein CLDN5 decreased in sensitive skin, compared to normal skin, suggesting a pathogenic role of TJ proteins in sensitive skin.^8^ This study found that sensitive skin displays low CLDN5 expression levels, disrupting the intercellular TJ structure. Importantly, *CLDN5* knockdown with siRNA reduced TEER in KC cultures, strongly supporting the crucial role of CLDN5 as a permeability barrier.

Previous studies have demonstrated that miRNAs regulate TJ protein expression in extracutaneous tissues.^22^, ^23^ The roles of miRNAs have been elucidated in various skin diseases. Cirilo et al.^24^ reported that miR‐195 plays an antiproliferative role in melanoma cells by targeting *PHB1*. Zhou et al.^25^ showed miR‐365 was involved in the development of cutaneous squamous cell carcinoma by targeting nuclear factor I/B. Zeng et al.^26^ found that miR‐143 may act as a potential preventive and therapeutic target in atopic dermatitis, which could inhibit inflammation by regulating *IL‐13Rα1* in epidermal KCs. However, the potential role of miRNAs in sensitive skin has not been elucidated yet. Our results suggest that miR‐224‐5p overexpression could be accountable, at least in part, for reduced CLDN5 expression in sensitive skin. RNA‐seq showed increased miR‐224‐5p expression in sensitive skin. Moreover, miR‐224‐5p decreased CLDN5 expression in KCs. Furthermore, miR‐224‐5p directly targets the 3`UTR of *CLDN5*, reducing its expression.

## 5 CONCLUSION

In conclusion, miRNA‐224‐5p overexpression causes CLDN5 deficiency, leading to a defective epidermal permeability barrier in sensitive skin. The modulation of miRNA‐224‐5p and/or CLDN5 expression could potentially be a therapeutic approach for sensitive skin management. Other mechanisms involved in downregulating CLDN5 expression in sensitive skin need to be explored to provide more evidence for the development of new therapeutic strategies.

## CONFLICT OF INTEREST STATEMENT

The authors declare no conflicts of interest.

## ETHICS STATEMENT

The study protocol was approved by the Ethics Committee of First Affiliated Hospital of Kunming Medical University. Written informed consent was obtained from all participants.

## Supporting information

Supporting Information

## Data Availability

All data generated or analyzed during this study are included in this article and its supplementary material files. Further inquiries can be directed to the corresponding author.
