# Supplementary material for: MiRNA‐224‐5p regulates the defective permeability barrier in sensitive skin by targeting claudin‐5
Source: Skin Res Technol. 2024 May 14;30(5):e13720. doi: 10.1111/srt.13720 (PMC11093069; doi:10.1111/srt.13720)
Supplement: Supplementary file 1 — Supporting Information [file SRT-30-e13720-s001.docx]

Table S1. Questionnaire for diagnosis of SS

| 1 | Would you say that your face/neck does not tolerate cold/hot weather or a cold/hot environment? |
| --- | --- |
| 2 | Would you say that your skin face/neck does not tolerate rapid temperature changes? |
| 3 | Have you already avoided the use of some cosmetic products that could, according to you, make your skin reactive? |
| 4 | Have you already had an adverse reaction on your face/neck to a cosmetic or hygiene product? |
| 5 | Would you say that your face/neck is reactive? |
| 6 | Have you already felt some itching, burning or tingling on your face/neck skin because of the wind or some cosmetics or hygiene products? |
| 7 | Is your face skin reactive to pollution, stress/emotions or menstrual cycle changes? |

Table S2. Primer and annealing tempertaure

| Gene | Forward | Reverse | Annealing tempertaure |
| --- | --- | --- | --- |
| ClLDN5 | CCTTCCTGGACCACAACATC | CCGAGTCGTACACTTTGCAC | 60°C |
| hsa-miR-224-5p | CAAGTCACTAGTGGTTCCGTT | GCTGTCAACGATACGCTACG | 60°C |
| hsa-miR-516b-5p | TCGAGGAGCTCACAGTCT | GCTGTCAACGATACGCTACG | 54°C |
| JAM2 | AGGCCAGCCCTCTAACACTT | TTCCAGAGTTGCCAGAAGAT | 61°C |
| hsa-miR-449c-5p | TGGAATGTAAGGAAGTGTGTGG | GCTGTCAACGATACGCTACG | 62°C |
| β-Actin | CCAGGGCGTTATGGTAGGCA | TTCCATATCGTCCCAGTTGGT | 60°C |
| U6 | CTCGCTTCGGCAGCACA | AACGCTTCACGAATTTGCGT | 59°C |

Table S3. siRNA sequences

| Name | Sequences |
| --- | --- |
| siRNA 1489 | 5′-UCUGCUGGUUCGCCAACAUTT-3′  5′-AUGUUGGCGAACCAGCAGATT-3′ |
| siRNA 1268 | 5′-GCAGUGCAAAGUGUACGACTT-3′  5′-GUCGUACACUUUGCACUGCTT-3′ |
| siRNA 1713 | 5′-GAUUGGCACGCUAUAUCGATT-3′  5′-AUUAUACACUUUGCACUGCTT-3′ |
| Negative control | 5′-UUCUCCGAACGUGUCACGUTT-3′  5′-ACGUGACACGUUCGGAGAATT-3′ |

Table S4. Top 20 differentially expressed miRNAs in 3 sensitive skin (SS) compared with normal skin tissue (N).

| Upregulated | | | Downregulated | | |
| --- | --- | --- | --- | --- | --- |
| Gene | P-value | Fold change  (SS vs. N) | Gene | P-value | Fold change  (SS vs. N) |
| hsa-miR-516b-5p | 7.66E-08 | 2.6211 | hsa-miR-202-5p | 9.01E-05 | -1.7703 |
| hsa-miR-449c-5p | 6.36E-08 | 2.1937 | hsa-miR-190a-3p | 0.001846 | -1.6387 |
| hsa-miR-224-5p | 1.93E-06 | 2.0595 | hsa-miR-376c-3p | 0.000181 | -1.6101 |
| hsa-miR-592 | 1.68E-06 | 1.9688 | hsa-miR-376b-5p | 0.002109 | -1.5985 |
| hsa-miR-519d-3p | 0.000446 | 1.8446 | hsa-miR-10a-3p | 0.000248 | -1.5419 |
| hsa-miR-511-5p | 1.87E-07 | 1.802 | hsa-miR-1185-5p | 0.000594 | -1.5317 |
| hsa-miR-508-5p | 6.32E-05 | 1.6948 | hsa-miR-101-5p | 0.000414 | -1.52 |
| hsa-miR-509-5p | 1.64E-06 | 1.6705 | hsa-miR-193a-3p | 1.19E-05 | -1.5043 |
| hsa-miR-517a-3p | 0.000396 | 1.6606 | hsa-miR-376b-3p | 0.003012 | -1.4789 |
| hsa-miR-506-3p | 0.000111 | 1.338 | hsa-miR-376a-3p | 0.001131 | -1.4329 |

*P*-values <0.05 were considered significant.
